# An Update on Inflamm-Aging: Mechanisms, Prevention, and Treatment

**DOI:** 10.1155/2016/8426874

**Published:** 2016-07-14

**Authors:** Shijin Xia, Xinyan Zhang, Songbai Zheng, Ramin Khanabdali, Bill Kalionis, Junzhen Wu, Wenbin Wan, Xiantao Tai

**Affiliations:** ^1^Department of Geriatrics, Shanghai Institute of Geriatrics, Huadong Hospital, Fudan University, Shanghai 200040, China; ^2^Department of Information, Chengdu Military General Hospital, Chengdu 610083, China; ^3^Department of Maternal-Fetal Medicine Pregnancy Research Centre and Department of Obstetrics and Gynaecology, University of Melbourne, Royal Women's Hospital, Parkville, VIC 3052, Australia; ^4^Department of Neurology, Zhongshan Hospital, Fudan University, Shanghai 200032, China; ^5^School of Acupuncture, Massage and Rehabilitation, Yunnan University of Traditional Chinese Medicine, Kunming 650500, China

## Abstract

Inflamm-aging is a challenging and promising new branch of aging-related research fields that includes areas such as immunosenescence. Increasing evidence indicates that inflamm-aging is intensively associated with many aging diseases, such as Alzheimer's disease, atherosclerosis, heart disease, type II diabetes, and cancer. Mounting studies have focused on the role of inflamm-aging in disease progression and many advances have been made in the last decade. However, the underlying mechanisms by which inflamm-aging affects pathological changes and disease development are still unclear. Here, we review studies of inflamm-aging that explore the concept, pathological features, mechanisms, intervention, and the therapeutic strategies of inflamm-aging in disease progression.

## 1. Introduction

Inflamm-aging [1] was first named by Franceschi et al. in 2000, and it is a new addition to the types of aging studies. Inflamm-aging plays an increasingly important role in the rate of aging and age-related diseases. Research in this area has attracted attention of scholars in many fields and significant progress has been made in the last decade. Here, we review the concept of inflamm-aging and describe various research strategies that have led to insights into its inflammatory characteristics and mechanisms of action. We also discuss the relationship of inflamm-aging with diseases and novel interventions to delay or prevent inflamm-aging-related diseases.

## 2. The Concept of Inflamm-Aging

A main feature of the aging process is a chronic progressive increase in the proinflammatory status, which was originally called “inflamm-aging” [1]. Subsequently other similar terms were used such as “inflammaging” [2], “inflamm-ageing” [3], and “inflammageing” [4]. Inflamm-aging is the expansion of the network theory of aging [5] and the remodeling theory of aging [6, 7]. The network theory of aging posits that aging is indirectly controlled by the network of cellular and molecular defense mechanisms. The remodeling theory, which was put forward to explain immunosenescence, is the gradually adaptive net result of the process of the body fighting malignant damage and is a dynamic process of optimization of the trade-off in immunity [6, 7]. In the process of aging, some researchers pointed out that the phenomenon where adaptive immunity declines is called immunosenescence, while the phenomenon where innate immunity is activated, coupled with the rise of proinflammation, is called inflamm-aging [8]. Some regard the chronic inflammatory process with age as inflamm-aging [9], while others proposed the oxidation-inflammation theory of aging [10]. Despite the lack of agreement on definitions and terminology, there is consensus that the primary feature of inflamm-aging is an increase in the body's proinflammatory status with advancing age. Furthermore, a new concept of “anti-inflammaging” was also proposed, which influences progressive pathophysiological changes, as well as lifespan, and acts along with inflamm-aging [11]. In the next section the characteristics of inflamm-aging are described in more detail.

## 3. The Inflammatory Characteristics of Inflamm-Aging

The five states of inflamm-aging are as follows [12]: low-grade, controlled, asymptomatic, chronic, and systemic. However, the inflammation during inflamm-aging is not in a controlled inflammatory state. We propose that inflammation in the process of inflamm-aging belongs to nonresolving inflammation [13]. Inflammation is a series of complex response events which are caused by the host system facing a pathogen infection or various types of tissue injury. These response events are characterized by interactions between the cells and factors in the microenvironment and by regulation of the balance between physiological and pathological signaling networks. In common conditions, inflammatory responses disappear when proinflammatory factors in infection and tissue injuries are eliminated and then change into a highly active and well regulated balanced state, which is called resolving inflammation [13]. However, in the presence of some as yet uncertain factors, such as persistent and low intensity stimulation and long-term and excessive response in target tissues, inflammation fails to move into a steady state of anti-infection and tissue injury repair; instead the inflammation continues and moves to a nonresolving inflammation state [13]. Given this background, inflammation in the process of inflamm-aging belongs to the state of nonresolving inflammation.

## 4. The Relationship between Inflamm-Aging and Diseases

Like the immune response, inflammation has a physiological function in the normal body. Moderate inflammatory response is beneficial to the body but when excessive, the response becomes harmful. Changes in the inflammatory cytokine network control the direction of the development of inflammation. The dynamic balance of the network of proinflammatory cytokines and anti-inflammatory cytokines maintains the physiologic function of inflammation in the normal body. Tipping the balance from anti-inflammation to proinflammation can lead to pathological changes. Persistent inflammation during the inflamm-aging process may cause inflammation-related diseases.

**Figure 1:**
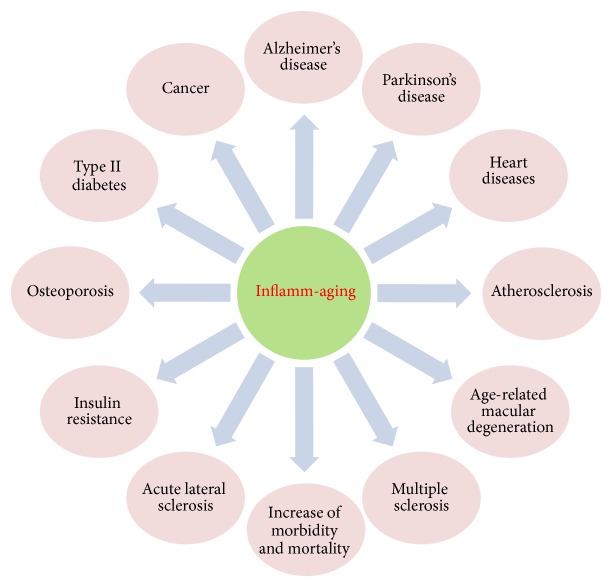
The relationship between inflamm-aging and diseases.

Inflamm-aging is a determinant of the speed of the aging process and of lifespan and is highly related to Alzheimer's disease [2], Parkinson's disease, acute lateral sclerosis, multiple sclerosis, atherosclerosis, heart disease, age-related macular degeneration [14], type II diabetes [15], osteoporosis and insulin resistance [16], cancer, and other diseases. Inflamm-aging also increases morbidity and mortality, significantly harming the health of patients, and causes a decline in the quality of life of patients [16]. Chronic, subclinical inflammation and immune disorders coexist in the process of inflamm-aging. Epidemiological studies show that with age there is an imbalance in the loss of old bone and the formation of new bone. Inflamm-aging may be one of the contributing factors to the imbalance and to the subsequent excessive loss of bone. Inflammatory markers of inflamm-aging provide clinicians with the necessary data for risk assessment of osteoporosis. Inflammatory cytokines may be therapeutic targets for improving the formation of bone in the elderly after bone operations [16]. Excessive inflammation during inflamm-aging increases the morbidity and mortality of patients after bone operations, even though the mechanism for this remains unclear [17]. In the process of inflamm-aging, the pathophysiological changes in the colon are revealed at the cellular and molecular levels, and these culminate in the inflammation that leads to injury of the gastric mucosa and epithelium as well as a decrease in the epithelium's ability to regenerate [18] (Figure 1).

However, inflamm-aging seems to be a double-edged sword in that it decreases immune function but also increases the autoreactivity of the body [17]. Inflamm-aging is beneficial to the body by neutralizing the harmful cytokines in the early stage of the life but has a detrimental role in the later life [17].

Unfortunately, the strong correlation between inflamm-aging and disease development is complex and unclear. Because immunosenescence and inflamm-aging coexist, it is difficult to distinguish whether the inflammation-related diseases are caused by inflamm-aging or immunosenescence. Moreover, the crucial question is whether there is a causal relationship between inflamm-aging and diseases, which needs integrated biological and clinical research to resolve.

## 5. The Mechanisms of Inflamm-Aging

While the mechanism of inflamm-aging is not completely understood, the current theories in the field are summarized below.

### 5.1. The Theory of Stress

Generally, stress is either beneficial or harmful to the body. During inflamm-aging, the body is constantly in the stress environment, which is caused by different kinds of stressors that induce and maintain the chronic proinflammatory status in the body. Stress, as one of regulated factors of immunity, provokes the greatest immune response in the bodies of young persons, whereas it provokes that weakest response in elderly persons with signs of immunosenescence and inflamm-aging [19, 20]. According to a series of studies involving different species from invertebrates to humans, from an evolutionary perspective, inflammation is closely related to innate immunity and stress [20]. Based on evolutionary studies, immune response, stress response, and inflammation form a defensive network in the body. However, the compatibility between inflammatory status and longevity and the paradoxical proinflammatory character in healthy centenarians strongly suggest the existence of physiological inflammation [21]. Therefore, inflammation and the proinflammatory status in healthy persons and centenarians show a beneficial response that helps the elderly deal with the stimuli generated by chronic antigen stressors [19]. However, excessive stress response, as well as an accompanying increasingly high proinflammatory response, leads to human inflamm-aging.

### 5.2. The Theory of Oxidation-Inflammation

There are close relationships between oxidative stress and inflamm-aging [21]. Based on the close relationship between oxidative stress, inflammation, and aging, the oxidation-inflammatory theory of aging (oxi-inflamm-aging) was proposed [10]. In this theory, oxidative stress leads to inflamm-aging and influences the homeostasis and health of the body. The relationship between the redox state and the function of immune cells influences the speed of aging and lifespan [10]. According to this theory, sufficient antioxidants in food may improve immune function, decrease oxidative stress, and extend the lifespan [22].

### 5.3. The Theory of Cytokines

Proinflammatory cytokines play an important role in inflamm-aging caused by chronic inflammation [23]. Type I cytokines (such as IFN-*γ* and TNF-*α*) and type II cytokines (IL-4) in unactivated and memory CD4+ T lymphocytes participate in the proinflammatory process [24]. Further research shows that CD8+ and CD4+ T lymphocytes play a pivotal role in the developing cytokine network, and this can lead to the chronic proinflammatory state and inflamm-aging [25]. In animal experiments, increased expression of IL-1*β*, IL-15, IL-18, TNF-*α* mRNA, and TNF-*α* protein in the peripheral blood of elderly horses appears to be a distinctive feature of inflamm-aging [26].

Elevated levels of IL-6 and TNF-*α* in the serum of the elderly are associated with disease, disability, and mortality [3]. Studies employing large patient cohorts provide evidence that the level of serum IL-6 is a reliable marker, or a predictive index, of inflamm-aging [3]. Experiments with healthy elderly people show that aging relates to increased proinflammatory status. The cause of the increased proinflammatory status is elevated levels of proinflammatory cytokines in the circulation including IL-1, IL-6, TNF-*α*, and PGE2 [27, 28]. Although the identity of cells that secrete proinflammatory mediators in elderly persons remains controversial, the prevailing view is that, during inflamm-aging, high levels of proinflammatory cytokines in the circulation create an inflammatory environment for tissues and organs [29]. However, differences in levels of IL-10 and TNF-*α* in individuals may play an important role in the final outcome of inflammation [29]. IL-6 and TNF-*α* are upregulated, while growth hormone and IGF-1 are downregulated in the process of aging. The overall balance of cytokines, such as IL-6 and TNF-*α*, appears to play a decisive role in aging. As well, genetic variations in the promoter regions of proinflammatory and regulated cytokine genes have effects on inflamm-aging and susceptibility to age-related diseases [29].

Pes et al. [30] found that the frequency of the -174C single nucleotide polymorphism (SNP) in the promoter region of IL-6 gene is increased in Italian male centenarians and the frequency of the -1082G SNP at the 5′ flanking region of the IL-10 gene coding sequence is increased among male centenarians. These data indicate that different alleles in different cytokine gene coding regions for pro- (IL-6) or anti-inflammatory (IL-10) cytokines may influence immune-inflammatory responses and individual lifespan expectancy, suggesting that inflammatory cytokine gene polymorphisms for immune system genes may regulate immune-inflammatory responses. Gene polymorphisms of proinflammatory cytokines associated with high levels of IL-6 have decreased capacity to reach extremely old age, whereas genotypes associated with high levels of IL-10 were increased in centenarians [30]. Genetic polymorphism in proinflammatory cytokine genes is necessary and has important consequences in the body. On the one hand, moderate levels of proinflammatory cytokines contribute to inducing a protective response, when the body is invaded by pathogens. On the other hand, excessive proinflammatory cytokines may cause immune-inflammatory diseases and even death. Indeed, the process of evolution has shaped the ability of the body to fight and control pathogens. Therefore, the proinflammatory response may be beneficial to the body in fighting potentially fatal infections. Thus, high levels of IL-6 and low levels of IL-10 are associated with enhanced ability against pathogens [30].

The vicious cycle of reciprocal causation between the proinflammatory cytokines and cellular senescence aggravates inflamm-aging. On the one hand, proinflammatory cytokines induce cellular senescence. Proinflammatory cytokines, such as TNF-*α*, IFN-*γ*, and IFN-*β*, induce cellular senescence in epithelial cells by producing reactive oxygen species and activating the ATM/P53/P21 (WAF1/Cip1) signaling pathway [31]. CXCR2, a chemokine receptor, induces cellular senescence of fibroblasts [32]. DNA damage produces proinflammatory cytokines (such as IL-1, IL-6, and IL-8) by activating the NF-*κ*B signaling pathway, blocking the cellular cycle and inducing and maintaining the phenotype of cellular senescence [32]. On the other hand, senescent cells secrete growth factors, proteases, chemokines, and cytokines such as IL-6 and IL-8 [33].

Most phenotypes of aging can be explained by an imbalance between proinflammation and anti-inflammation, which results in inflamm-aging with a low chronic proinflammatory status. However, centenarians have high levels of inflammatory mediators and more anti-inflammatory cytokines, suggesting that inflamm-aging can coexist with longevity, even though the underlying mechanisms have not been uncovered [34].

### 5.4. The Theory of DNA Damage

Sustained telomere DNA and mitochondrial DNA damage, caused by exogenous and endogenous factors, can induce errors of DNA replication or translation, which leads to point mutations or chromosomal rearrangements and stress reactions via various signaling pathways, which eventually contributes to cellular senescence. Researchers found that, in human senescent primary cells, the shortest telomeres lack most of the telomere repeat sequence, which leads to DNA damage accumulation and terminal cell cycle arrest and further induces replicative senescence [35]. A persistent DNA damage response (DDR) caused by telomere shortening is a key mechanism involved in replicative senescence and aging process [36]. New evidence indicates that DNA damage response (DDR) signaling is a major link between cell senescence and organism aging. DDR activation of senescent cells contributes to an increase in the proinflammatory secretory phenotype (PSP), which in turn triggers the activation of adjacent cell DDR and PSP. This local inflammatory environment eventually becomes systemic. The increasing number of cells with DDR activation may exacerbate inflamm-aging [37]. These results suggest that cells in an inflammatory environment induce aging at the systemic level. Stem cells and stromal fibroblasts differentiate into proinflammatory cytokine overexpressing cells and consequently the cytokine network breaks down, inducing inflamm-aging as a result of the accumulation of DNA damage [38]. Proinflammatory cytokines in the microenvironment of cells with DNA damage further induce inflamm-aging. Macrophages, which mediate the main effects of inflamm-aging, amplify inflamm-aging self-propagation via a cascade effect on the local and systemic proinflammatory response [38].

### 5.5. The Theory of Autophagy

Autophagy plays an important role in stress, removing harmful substances in cells to maintain homeostasis and normal metabolism [39, 40]. Autophagy transfers the abnormal substances of the cell to lysosomes for degradation and also plays a role in many pathophysiological processes [41]. For example, autophagy plays important roles in removing abnormal proteins, adapting to hunger, and cancer. More and more evidence shows that autophagy is important in increasing longevity. For example, knocking out the autophagy gene Atg7 leads to the accumulation of proteins and organelles in the cell, causing cellular senescence [42].

The process of aging accompanies disorder in homeostasis. However, autophagy plays an important role in maintaining homeostasis and delaying aging. In the process of aging, autophagic cleansing capacity declines gradually, which induces mitochondrion disordering and protein accumulation. This leads to increased reactive oxygen species (ROS) and consequently oxidative stress. Destabilized lysosomes release ROS, which activate Nod-like receptor 3 (NLRP3), and this initiates an inflammatory cascade reaction. During this process, inactive precursors of IL-1*β* and IL-18 are increased, and IL-1*β* and IL-18 release is stimulated, which causes an inflammatory reaction and accelerated aging [43].

### 5.6. The Theory of Stem Cell Aging

Stem cell aging is closely related to inflammation [44]. Stem cell aging is the cellular basis of aging and chronic inflammation is one of the main factors that induces stem cell aging. In the chronic inflammatory process, proinflammatory factors activate NF-*κ*B/MAPKs, TOR, RIG-I, and JAK/STAT signaling pathways to induce cells to synthesize and to secrete large amounts of inflammatory cytokines, such as TNF*α* and IL-1*β* [45], which leads to a chronic low degree of inflammation in the environment of cells, thereby inhibiting the regenerative capacity of stem cells. This leads to dysfunctional differentiation of stem cells, damage of the stem cell microenvironment (i.e., that stem cell niche), homeostasis, and stem cell aging [44].

## 6. Regulatory Signaling Pathways of Inflamm-Aging

In principle, the pathways controlling inflammation are potential regulatory signaling pathways of inflamm-aging. The NF-*κ*B and TOR signaling pathways, in particular, have been investigated.

### 6.1. NF-*κ*B Signaling Pathway

NF-*κ*B, a nuclear transcription factor, is regarded as the main molecular switch of inflammatory pathways. The NF-*κ*B signaling pathway may also regulate inflamm-aging [8]. However, the longevity gene, SIRT1, can be combined with a subunit of NF-*κ*B, Rel/p65, to deacetylate K310 and inhibit the transcriptional activity of NF-*κ*B [46, 47]. NF-*κ*B can regulate the occurrence of aging, whereas SIRT1 may regulate NF-*κ*B to delay aging [48]. Thus, NF-*κ*B can regulate both aging and inflammation [49]. NF-*κ*B can also inhibit inflammatory reactions by regulating SIRT1 (Sir2 homolog) and FoxODAF-16 [8].

### 6.2. TOR Signaling Pathway

TOR, a highly conserved serine/threonine protein kinase, plays an important role in the regulating growth and proliferation of cells [50]. According to its different functions, TOR can be divided into TORC1 and TORC2. The former is sensitive to rapamycin, participating in the biological process of transcription and translation in cells. The latter is insensitive to rapamycin and mainly regulates remodeling the cytoskeleton [51]. The TOR signaling pathway regulates longevity. When TOR signaling is decreased or inactivated, the lifespan of wireworms and* Drosophila* is extended. Similarly, the lifespan of yeast can be increased by exposure to a low dose of rapamycin [52]. At present, it is believed that TORC1 participates in regulating aging. S6K is a positive regulation target of TORC1. In the mouse knockout of the S6K gene, lifespan is extended. 4E-BP is a gene that is necessary to lifecycle and is a negative regulator of TORC1. When 4E-BP is overexpressed, lifespan is prolonged [53].

In terms of the physiological function of TOR signal regulation, TOR regulates growth during embryonic development, and in maturity TOR regulates metabolism. However, in old age TOR signaling regulation is excessively activated and is associated with many age-related diseases. The excessive production of cytokines and inflammatory factors induces aging and the changes to the local microenvironment, causing age-related diseases. TOR regulates inflamm-aging by activating NF-*κ*B [54].

### 6.3. RIG-I Signaling Pathway

Retinoic-acid-inducible gene-I (RIG-I) may be involved in inflamm-aging. RIG-I is induced via the ataxia telangiectasia mutated interferon regulatory factor-1 (ATM-IRF1) axis in senescent cells and interacts with increased levels of IL-6 and IL-8. The activation of RIG-I signaling pathway upregulates IL-6 expression [55]. RIG-I is a caspase recruitment domain- (CARD-) containing protein that functions as a cytoplasmic RNA sensor [55]. Liu et al. [55] showed that IL-6 and IL-8 levels increase in replicating senescent cells. They reported that senescent cells transfected with RIG-I show increased secretion of IL-6. However, knockdown of RIG-I in senescent cells leads to the extension of the lifespan of cells, which shows that RIG-I-induced inflammation plays a role in promoting and maintaining aging. Interfering with RIG-I expression significantly decreases the levels of inflammatory cytokines in senescent cells [55]. This imbalance in the inflammatory process may cause chronic inflammation during aging.

### 6.4. Notch Signaling Pathway

The Notch signaling pathway is a major intercellular communication pathway that is highly conserved through evolution. Notch signaling plays an essential role in aging [56]. At the cellular level, aging of vascular endothelial cells (EC) leads to senescence. Senescent EC secrete proinflammatory cytokines and this is often accompanied by a low-grade chronic upregulation of certain proinflammatory responses [56]. Constitutive activation of Notch signaling induces EC senescence. Consistent with these results, HeyL, a Notch downstream target, is elevated in aged compared to young EC. Notch activation also triggers EC inflammatory responses by upregulating expression of a panel of proinflammatory cytokines/chemokines and adhesion molecules in EC. This has revealed a novel function of Notch1 signaling in EC biology and may shed light on the mechanism whereby Notch signaling may contribute to some age-related vascular diseases characterized by chronic inflammation.

### 6.5. Sirtuin Signaling Pathway

Silent information regulator (Sir) proteins regulate lifespan in multiple model organisms [57]. Sir2 (SIRT1–7 in mammals) is a NAD-dependent deacetylase that has been implicated in aging and inflammation in yeast, worms, and flies. SIRT1, the most extensively studied in mammals, has a highly conserved NAD-dependent sirtuin core domain and is a good candidate lifespan regulator along with the other six homologs. Recent studies showed that SIRT1 is a potent anti-inflammatory protein and inhibits the COX-2/MMP pathway via suppression of the potent proinflammatory factor NF-*κ*B. NF-*κ*B signaling is limited by SIRT6, which is recruited to NF-*κ*B target gene promoters by a physical interaction with the NF-*κ*B subunit RelA. SIRT6 deacetylates histone H3 lysine 9 on target gene promoters, thereby altering the chromatin structure to facilitate NF-*κ*B destabilization and signal termination. SIRT1 activation decreases the proinflammatory effects induced by TNF-*α*. In addition, treatment with SIRT1 activators such as resveratrol, or overexpression of SIRT1, inhibits the expression and activation of the main proinflammatory regulator NF-*κ*B, which is increased by TNF-*α*. When SIRT1 is overexpressed, the anti-inflammatory action of SIRT1 is similar to that exerted by resveratrol. Resveratrol, as an SIRT1 activator, inhibits TNF-*α*-induced inflammatory factor release. Resveratrol effectively inhibits the activation of proinflammatory factors by activating SIRT1, leading to the deacetylation of NF-*κ*B p-65 and subsequent downregulation of TNF-*α*-induced COX-2 and MMP expression [58].

### 6.6. TGF-*β* Signaling Pathway

Sequence variations in a variety of pro- or anti-inflammatory cytokine genes have been found to influence successful aging and longevity. TGF-*β*1 has been shown to have an essential role in inflammation and in the maintenance of immune response homeostasis. TGF-*β*1 belongs to the group of cytokines with anti-inflammatory effects and is a deactivating factor of macrophages with potent anti-inflammatory properties. Because of the role played by the transforming growth factor *β*1 (TGF-*β*1) in inflammation and the regulation of immune responses, variability of the TGF-*β*1 gene may affect longevity by playing a role in inflamm-aging. The potential role of TGF-*β*1 in aging and longevity has been suggested by many in vitro and in vivo studies. In particular, TGF-*β*1 gene overexpression has been observed in human fibroblasts that display a senescent-like phenotype following exposure to oxidative stress, which may help to seek a new proposal to treat disease-related aging [59].

### 6.7. Ras Signaling Pathway

Ras is an important signaling molecule involved in atherogenesis and is a proinflammatory molecule involved in inflammation and aging. Ras promotes aging in yeast and cellular senescence in primary human fibroblasts. Activation of Ras drastically increases the expression of proinflammatory cytokines, in part through extracellular signal-regulated kinase activation. Introduction of Ras into arteries enhances vascular inflammation and senescence [60].

Ang II promotes reactive oxygen species (ROS) production, cell growth, apoptosis, cell migration and differentiation, and extracellular matrix remodeling. Ang II regulates gene expression and can activate multiple intracellular signaling pathways leading to tissue injury. Ang II also mediates several key events in the inflammatory process. Blocking Ang II signaling protects against neurodegenerative processes and promotes longevity in rodents. Ang II-induced ROS production via the AT1 receptor promotes the onset of vascular senescence associated with functional and structural changes to blood vessels that contribute to age-related vascular diseases [60].

**Figure 2:**
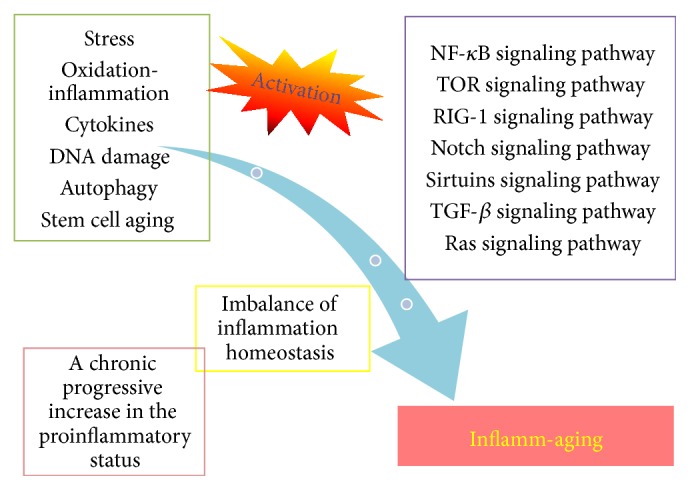
The mechanisms and regulated signaling pathways of inflamm-aging.

At present, the mechanisms of inflamm-aging remain unclear, because the methods and tools used for research into the mechanisms of inflamm-aging are not sufficiently sophisticated to explain inflammatory reactions caused by complex inflammatory cytokine cascades during inflamm-aging. Unfortunately, an adequate and reliable assessment system for aging has not yet been established. Furthermore, the causal relationships between inflammation and aging have not yet been elucidated. Moreover, the mechanisms referred to above need to be further verified. Inflamm-aging influences all levels of function, from cells to tissues, organs, and the whole body. Inflamm-aging also involves aberrant gene regulation and an imbalance in energy metabolism as well as interactions between these two factors. The mechanisms of inflamm-aging are very complicated and require multidisciplinary research to further investigate the interactions at multiple levels from cells to the whole body (Figure 2 and Table 1).

## 7. Potential Markers of Inflamm-Aging

One of the constraining factors of aging research is the lack of recognized, accurate, and reliable biological markers. The main biological markers of aging can be categorized as follows: (1) the marker is related to age; (2) the marker does not change with disease; (3) the marker does not change with metabolic and nutrient conditions; (4) the marker is influenced by the process of aging; (5) the marker does not change in immortalized cells. Unfortunately, the biological markers of aging have not yet been defined and need to be further investigated. This will facilitate the evaluation of the degree of inflamm-aging and assist in identifying the molecular mechanisms underlying inflamm-aging. The potential markers of inflamm-aging may include* immune cell* markers,* serum cytokine* markers, and microRNAs.

### 7.1. Immune Cell Markers

A main characteristic of the immune system in the elderly is antigenic T cell accumulation. The shortage of naive CD8+ T lymphocytes is regarded as a reliable biological marker related to the risk of death. The increase in CD8+ T cells, a decrease in CD4+ T cells and CD19+ B cells, and inhibition of mitogen-induced proliferation of T cells may be predictors of inflamm-aging [3].

### 7.2. Serum Cytokine Markers

The increase in serum IL-6 in the elderly is related to diseases, disability, and mortality. A study on a large cohort showed that IL-6 in the serum is a reliable marker of disability in the elderly and contributes to the predictive index of disability and mortality [3]. The functions of some cytokines, such as IL-10 and TNF-*α*, are complex and play opposing roles in the systemic inflammatory reaction. IL-10 inhibits inflammatory reactions, while TNF-*α* activates inflammatory reaction locally and systemically. Different regulation of IL-10 and TNF-*α* may be essential to the final outcome of the inflammatory reaction. Therefore, levels of IL-6, TNF-*α*, and IL-10 may be regarded as serum markers of inflamm-aging [3].

### 7.3. MicroRNA Markers

MicroRNAs (miRs) are a class of molecules involved in the regulation of gene expression and are emerging as modulators of biological pathways including NF-*κ*B, mTOR, sirtuins, TGF-*β*, and Wnt. miRs may be associated with inflammation, cellular senescence, and age-related diseases and they can be classified as inflammation-associated (inflamm-miRs) and senescence-associated (SA-miRs) [61]. miR-based anti-inflammatory mechanisms may play a crucial role during aging, where a chronic, low-level proinflammatory status is likely sustained by the cell senescence secretome and by progressive activation of immune cells over time. Circulating miRs seem to be promising biomarkers of major age-related diseases [60]. Some miRs are found in plasma and leukocytes in centenarians. Some miRs, such as miR-21, miR-126, and miR-146a, which target mRNAs belonging to the NF-*κ*B pathway can be considered as both SA-miRs and inflamm-miRs [61]. Thus specific inflamm-miRs may be regarded as biomarkers of inflamm-aging [62].

## 8. Intervention in Inflamm-Aging

A significant feature of inflamm-aging is that there is a chronic, low-grade, microinflammatory state in the body. Therefore, drugs used for the treatment of inflamm-aging must be effective, safe, nontoxic, and appropriate for long-term use. Calorie restriction, zinc (Zn), resveratrol,* Epimedium* total flavonoids, and icariin have these characteristics and may be candidate drugs to treat inflamm-aging.

### 8.1. Calorie Restriction

Calorie restriction (CR), also known as dietary restriction (DR), has been regarded as the gold standard of many aging interventions to counteract aging. CR together with adequate nutrient intake prolongs maximum lifespan possibly through beneficial metabolic, hormonal, and functional alterations [63]. The antiaging action of CR may be based largely on its ability to suppress oxidative stress related alterations and oxidative stress induced age-related diseases [64, 65]. CR can modulate many important inflammatory signaling pathways involved in aging and inflammation such as NF-*κ*B, mTOR, and MAPK [66]. The age-related upregulation of NF-*κ*B, IL-*β*, IL-6, and TNF-*α* in the proinflammatory states of the aging process is attenuated by CR [67].

### 8.2. Zn

Zn is an especially important, necessary microelement for the human body and has an important impact on regulating the balance between the genetic expression of metalloproteinases (MPs) and MPs inhibitors, on maintaining inducible nitric-oxide synthase (iNOS) activity, and on many biochemical functions. The interaction between Zn and IL-6, TNF-*α*, or heat shock protein 70 (Hsp70) regulates the immune-inflammatory reaction. The elderly frequently lack adequate levels of Zn. A moderate amount of Zn added to the diet may expand the lifespan of the elderly, which suggests that Zn may intervene in inflamm-aging [68].

### 8.3. Resveratrol

Some studies have found that resveratrol affects aging and lifespan in mammals [22]. Researchers reported that this potent natural compound is a SIRT1 activator and may help in preventing the aging-related decline in heart function and neuronal loss through stimulating SIRT1 activation [69]. Moreover, resveratrol decreases ovarian inflammation via inhibition of NF-*κ*B by upregulation of PPAR-*γ* and SIRT1 expression [70]. Furthermore, resveratrol can suppress tumorigenesis at least in part by targeting Sirt1 and suppressing NF-*κ*B activation [71]. Resveratrol suppresses the upregulation of proinflammatory molecules (such as IL-1*β* and IL-6) by TNF-*α* in 3T3 cells in a dose-dependent manner. However, knockdown of Sirt1 by RNA interference makes 3T3 cells susceptible to TNF-*α* stimulation and decreases the anti-inflammatory effect of resveratrol. The potential anti-inflammatory mechanisms of resveratrol involve a reduction in NF-*κ*B subunit RelA/p65 acetylation, which is notably Sirt1 dependent. Resveratrol also attenuates the phosphorylation of the mammalian target of rapamycin (mTOR) and S6 ribosomal protein (S6RP) while ameliorating inflammation [72]. These data demonstrate that resveratrol may have inhibitory effect on inflamm-aging.

### 8.4. Epimedium Total Flavonoids and Icariin

In our previous studies, based on the neuroendocrine-immunological network, we used an inflammatory cytokine and genetic receptor chip-based assay to detect critical genes in the hippocampus, hypothalamus, hypophysis, adrenal gland, and spleen of elderly rats. We also detected the proteins corresponding to the genes referred to above. The findings showed that overexpression of some proinflammatory cytokines at the transcription and protein level may be involved in the highly proinflammatory reactive state during inflamm-aging. Additionally, our experiments showed that* Epimedium* total flavonoids (EF) and icariin (Ica) reduced the proinflammatory response, enhanced the anti-inflammatory response, and reestablished the equilibrium between proinflammatory and anti-inflammatory reactions in the process of inflamm-aging [73, 74].

### 8.5. Metformin

The biguanide drug metformin, a type of hypoglycemic drug, is widely prescribed to treat type 2 diabetes and metabolic syndrome. Researchers have also noted the effect of metformin on delaying aging, an effect validated in rodents [75] and in the nematode* Caenorhabditis elegans* [76]. Recently, Hall conducted a clinic trial called “Metformin, Anti-aging,” which was supported by the U.S. Food and Drug Administration (FDA) [77]. This was a landmark event in the history of aging research and showed that metformin may be used as an antiaging drug to improve the health span of humans. The mechanisms underlying the antiaging effects of metformin remain unclear. Several lines of evidence support that metformin may act by inducing metabolism associated with dietary restriction (DR) to increase lifespan and thereby limit the onset of age-associated diseases across species [78, 79]. A potential mediator of metformin benefits is the AMP-activated kinase (AMPK) and metformin can be viewed as DR-like compound [76]. Cabreiro et al. reported that metformin disrupts the bacterial folate cycle, leading to reduced levels of S-adenosylmethionine (SAMe) and decelerated aging in* C. elegans* [80]. Meanwhile, Moiseeva et al. showed that metformin inhibits the expression of genes coding for multiple inflammatory cytokines seen during cellular senescence, and metformin blocks the activity of NF-*κ*B [81]. The effects of metformin on anti-inflammation and antiaging imply its potential on inflamm-aging.

## 9. Novel Research Strategies in Inflamm-Aging

Research into inflamm-aging is still at an early stage. The mechanisms, biomarkers, evaluation method, research models, and intervention methods of inflamm-aging have not been fully elucidated. Moreover, inflamm-aging involves cells, organs, and the whole body and this requires an extensive and varied range of research investigations.

Based on the essential effects and our understanding of inflammatory cytokine pathways in the process of inflamm-aging, we can begin to explore the inflammatory cytokine network and perform a quantitative evaluation of inflamm-aging. Inflammatory cytokines, including interleukins, tumor necrosis factor, and interferon, mediate their effects by binding to their receptors and competing in a complex cell-cell network. These cytokines act in both paracrine and autocrine ways to exert direct effects on the microenvironment. This plays an important regulatory role by activating inflammatory and immune cells and by releasing cytokines. In addition, inflammatory cytokines induce the systemic inflammatory response in the circulation. Interactions between many inflammatory cytokines comprise the inflammatory cytokine network, which features polyphyletism, pleiotropy, and overlapping effects. Inflammatory cytokines form a complex network which extends in all directions and throughout the whole body. The inflammatory cytokine network can be divided into the proinflammatory cytokine network and anti-inflammatory cytokine network. As with the immune reaction, the inflammatory reaction is also a normal defense function. A moderate inflammatory reaction is advantageous to the body, whereas a high reaction is harmful and the outcome of these reactions is determined by changes in the inflammatory cytokine network. The dynamic balance between the proinflammatory cytokine network and the anti-inflammatory cytokine network maintains the normal function of inflammation in body. Once the balance is broken, pathological inflammation occurs [82, 83]. Therefore, we infer that the cause of inflamm-aging is an imbalance in the proinflammatory cytokine and anti-inflammatory cytokine networks, which leads to a proinflammatory status with increasing age. This may be the mechanism of inflamm-aging.

The changes in the inflammatory cytokine network have three characteristics: parallelity, multilevel action, and nonlinearity. Unraveling the inflammatory cytokine network will require nonlinear research methods such as systems biology methods. The inflammatory cytokine network is a typical systems biology issue, which needs to replace the model involving individual genes with a systems biology model.

Systems biology is an interdisciplinary field integrating multiple disciplines such as biology, medicine, mathematics, physics, and chemistry. It integrates many kinds of experimental data and biological information to build a mathematical model tested and verified by experimental data and finally predicts the behavior of biological systems [84]. Systems biology provides an excellent opportunity to elucidate the essential features of the inflammatory cytokine network. It also provides a theoretical guide and new ways to illustrate the relevant mechanisms and build a quantitative evaluation system for the inflammatory cytokine network, which may provide a breakthrough in research on inflamm-aging.

Aging (including inflamm-aging) is also a systems biology issue involving a complex process that results from the combined effects of many factors. Cellular senescence is the basic unit of biological aging. Organ aging is not only the macropresentation of cellular senescence but also a bridge that connects cellular senescence and integral aging. Cellular senescence, organ aging, and integral aging form a chain of aging, and cellular senescence is the critical link in the chain of aging. In the aging process, at all levels (i.e., genes, proteins, metabolites, cells, and tissues), the organism undergoes varying degrees of change in these structures, such that the body's systems (e.g., the nervous system, endocrine system, cardiovascular system, respiratory system, digestive system, urinary system, and motor system) undergo a significant functional decline. Aging is not the result of a unilateral factor, but a systemic decline in body function, and the distinctive features of aging are systemic [85]. The systemic features of aging strongly reflect the gradual changes in body function. Functional changes in the process of aging appear gradually with age, and all the changes are the results of progressive accumulation; time is the driving factor. The body is composed of various tissues and organs, so aging is a gradual process, not a point but an evolution of tissues and organs over time.

Multidisciplinary research including the fields of medicine, biology, mathematics, computer science, and systems biology should be applied and developed to investigate the mechanisms of inflamm-aging and the relationship between inflamm-aging and age-related diseases. Specifically, the important scientific problems that need to be addressed are the following: (1) the regulatory mechanisms responsible for the development of inflamm-aging and (2) the molecular mechanisms, the regulatory network, and the key role of the transformation from inflammation to age-related diseases in the process of inflamm-aging.

In summary, inflamm-aging and the inflammatory cytokine network are both classical systems biology issues. The inflammatory cytokine network is involved in the process of inflammation and senescence and may be the ideal breakthrough point of research into inflamm-aging. Omics, such as genomics, transcriptomics, proteomics, and metabolomics, are excellent methods to solve systemic biology problems. Therefore, under the guidance of systems biology, it would be novel strategy to conduct basic research into inflamm-aging using omics methods to identify characteristic inflammatory cytokine genes in the process of aging and to uncover new mechanisms to regulate inflammatory cytokines during inflamm-aging. This will also illustrate the mechanism of inflamm-aging and provide new ways to assess inflamm-aging.

## 10. A Novel Concept of Immuno-Inflamm-Aging

There is an essential relationship between inflammation and immunity. Both the inflammatory steady state and immune steady state have defense functions, protecting the body from injury [86]. However, when the inflammatory steady state and immune steady state are broken, excessive inflammation and pathological immunity ensue and the normal physiological function of the body is compromised, which causes immune-inflammatory diseases. Inflammation and immunity coexist in the same pathological process, the two sides of a whole, and are inseparable. In a sense, inflammatory cells are immune cells [87]; therefore, they have the same cellular foundation. Many inflammatory and immune cells have the same cytokine receptors [88], which mediate cell-cell and cell-cytokine interactions. The internal relationship between inflammation and immunity is not known, and the causes and pathological mechanisms of immune-inflammatory diseases are not understood and this limits effective treatments for immune-inflammatory diseases [86].

EF and Ica, anti-inflammatory immunomodulatory Chinese traditional medicines, not only reduce inflammation but also regulate immunity. As anti-inflammatory, immunomodulatory Chinese traditional medicines, EF and Ica have the double effects of intervening in immunosenescence and inflamm-aging, suggesting an intimate relationship between immunosenescence and inflamm-aging [73, 74].

Based on the integrated relationship between oxidative stress and inflammatory stress, De La Fuente and Miquel proposed an innovative oxidation-inflammation theory of aging (oxi-inflamm-aging) [10]. The oxi-inflamm-aging theory posits that chronic oxidative stress affects all immune cells, and particularly regulatory systems such as neural, endocrine, and immune systems, as well as the mutual interactions among these systems. These events lead to stable, internal environment disorders that are harmful to health. The relationship between the redox state and immune function affects the speed of aging and lifespan. A diet with sufficient antioxidants improves immune function, reduces oxidative stress, and prolongs lifespan, which supports the notion that the inflammatory response of immune cells and the immune system play an important role in oxi-inflamm-aging [10]. Inflamm-aging and immunosenescence are connected, and they cause and affect each other. This can result in a vicious cycle that further aggravates the occurrence and development of age-related diseases including atherosclerosis, metabolic syndrome, type 2 diabetes, insulin resistance, osteoporosis, bone arthritis, muscle mass and muscle weakness, cancer, and neurodegenerative diseases.

It is currently not possible to distinguish whether diseases are caused by inflamm-aging alone or immunosenescence alone [30, 89]. Therefore, we propose that inflamm-aging is accompanied by immunosenescence, and they occur together. We propose the novel concept of immune/inflammatory aging (immuno-inflamm-aging), instead of the individual concepts of inflamm-aging and immunosenescence.

## Figures and Tables

**Table 1:** Inflamm-aging-related mechanisms and regulated signaling pathways.

Mechanisms	Effects	Signaling pathways	References
Stress	Chronic antigen stressors lead to excessive stress response and contribute to inflamm-aging	Ras	[19, 20]
Oxidation-inflammation	Oxidative stress and inflammation influences the homeostasis and health of body	NF-*κ*B, Notch, TGF-*β*, sirtuin	[10]
Cytokines	High levels of proinflammatory cytokines result in inflamm-aging and age-related diseases	mTOR, RIG-I, Notch	[29, 30, 52, 53]
DNA damage	DNA damage response increases proinflammatory cytokines	NF-*κ*B	[36]
Autophagy	Autophagic function dysfunction leads to increased oxidative products and oxidative stress	NF-*κ*B	[40, 41]
Stem cell aging	Chronic inflammation induces stem cell aging and inhibits the regenerative capacity of stem cells	NF-*κ*B, mTOR, RIG-I	[42]
